# (2*E*)-*N*′-[(*E*)-Benzyl­idene]-3-phenyl­prop-2-enohydrazide from synchrotron radiation

**DOI:** 10.1107/S1600536812028504

**Published:** 2012-06-30

**Authors:** Samir A. Carvalho, Edson F. da Silva, Carlos A. M. Fraga, Solange M. S. V. Wardell, James L. Wardell, Edward R. T. Tiekink

**Affiliations:** a FioCruz-Fundação Oswaldo Cruz, Instituto de Tecnologia em Fármacos-Farmanguinhos, Rua Sizenando Nabuco, 100, Manguinhos, 21041-250 Rio de Janeiro, RJ, Brazil; b Programa de Pós-Graduação em Química, Instituto de Química, Universidade Federal do Rio de Janeiro, 21949-900 Rio de Janeiro, RJ, Brazil; c Laboratório de Avaliação e Síntese de Substâncias Bioativas, Faculdade de Farmácia, Universidade Federal do Rio de Janeiro, PO Box 68023, 21941-902 Rio de Janeiro, RJ, Brazil; d CHEMSOL, 1 Harcourt Road, Aberdeen AB15 5NY, Scotland; e Centro de Desenvolvimento Tecnológico em Saúde (CDTS), Fundação Oswaldo Cruz (FIOCRUZ), Casa Amarela, Campus de Manguinhos, Av. Brasil 4365, 21040-900 Rio de Janeiro, RJ, Brazil; f Department of Chemistry, University of Malaya, 50603 Kuala Lumpur, Malaysia

## Abstract

In the title compound, C_16_H_14_N_2_O, the dihedral angle between the phenyl rings is 25.48 (12)°. An *E* conformation is found for each of the imine [1.269 (3) Å] and ethyl­ene [1.313 (3) Å] bonds. In the crystal, mol­ecules are linked by N—H⋯O hydrogen bonds, leading to zigzag chains along [010]. Supra­molecular layers in the *ab* plane are formed, whereby the chains are linked by C—H⋯N and C—H⋯π inter­actions.

## Related literature

For the biological activity of (*E*)-cinnamoylhydrazone derivatives against Chagas’ disease, see: Carvalho *et al.* (2012*b* ▶). For background to Chagas’ disease, see: Rassi *et al.* (2010 ▶); Soeiro & de Castro (2011 ▶). For related structural studies, see: Carvalho *et al.* (2009 ▶, 2010*a* ▶,*b* ▶, 2012*a* ▶).
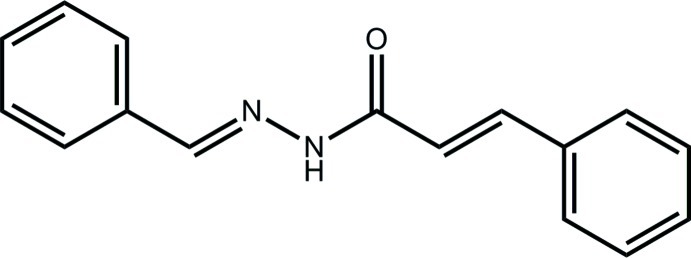


## Experimental

### 

#### Crystal data

C_16_H_14_N_2_O
*M* *_r_* = 250.30
Orthorhombic, 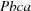

*a* = 11.473 (19) Å
*b* = 7.507 (13) Å
*c* = 30.50 (5) Å
*V* = 2627 (8) Å^3^
*Z* = 8
Synchrotron radiation
λ = 0.6943 Å
μ = 0.04 mm^−1^
*T* = 120 K
0.12 × 0.03 × 0.02 mm

#### Data collection

Bruker SMART APEXII CCD diffractometer
14542 measured reflections
1876 independent reflections
1442 reflections with *I* > 2σ(*I*)
*R*_int_ = 0.084
θ_max_ = 22.7°

#### Refinement

*R*[*F* ^2^ > 2σ(*F* ^2^)] = 0.049
*wR*(*F* ^2^) = 0.140
*S* = 1.05
1876 reflections
175 parameters
1 restraint
H atoms treated by a mixture of independent and constrained refinement
Δρ_max_ = 0.18 e Å^−3^
Δρ_min_ = −0.20 e Å^−3^

### 

Data collection: *APEX2* (Bruker, 2004 ▶); cell refinement: *SAINT* (Bruker, 2004 ▶); data reduction: *SAINT*; program(s) used to solve structure: *SHELXS97* (Sheldrick, 2008 ▶); program(s) used to refine structure: *SHELXL97* (Sheldrick, 2008 ▶); molecular graphics: *ORTEP-3* (Farrugia, 1997 ▶) and *DIAMOND* (Brandenburg, 2006 ▶); software used to prepare material for publication: *publCIF* (Westrip, 2010 ▶).

## Supplementary Material

Crystal structure: contains datablock(s) global, I. DOI: 10.1107/S1600536812028504/hb6861sup1.cif

Structure factors: contains datablock(s) I. DOI: 10.1107/S1600536812028504/hb6861Isup2.hkl

Supplementary material file. DOI: 10.1107/S1600536812028504/hb6861Isup3.cml

Additional supplementary materials:  crystallographic information; 3D view; checkCIF report

## Figures and Tables

**Table 1:** Hydrogen-bond geometry (Å, °) *Cg*1 is the centroid of the C1–C6 benzene ring.

*D*—H⋯*A*	*D*—H	H⋯*A*	*D*⋯*A*	*D*—H⋯*A*
N2—H2n⋯O1^i^	0.88 (2)	1.93 (2)	2.816 (6)	175 (2)
C5—H5⋯N1^ii^	0.95	2.57	3.433 (7)	151
C3—H3⋯*Cg*1^iii^	0.95	2.92	3.618 (7)	131
C6—H6⋯*Cg*1^iv^	0.95	2.75	3.645 (7)	158

Symmetry codes: (i) 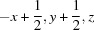; (ii) 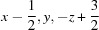; (iii) 
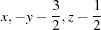
; (iv) 
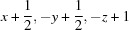
.

**Fig. 1.**
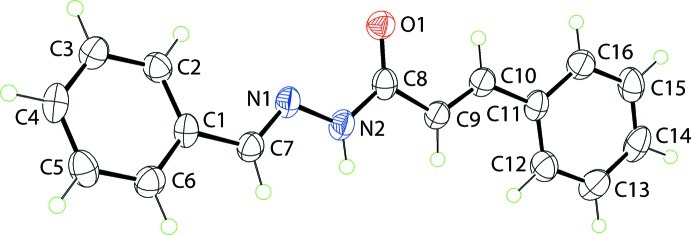
The molecular structure of (I) showing displacement ellipsoids at the 50% probability level.

**Fig. 2.**
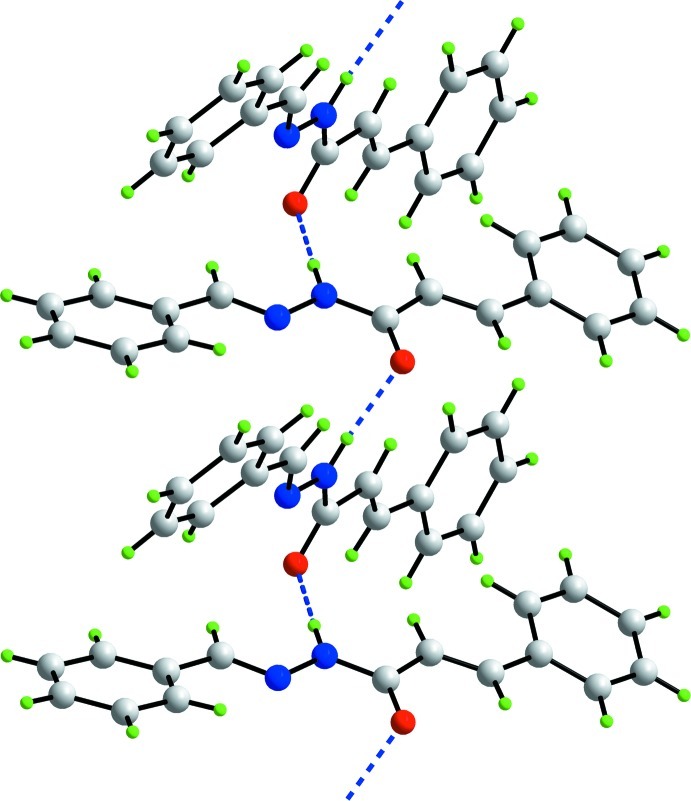
A view of the supramolecular zigzag chain along the b axis in (I). The N—H···O hydrogen bonds are shown as blue dashed lines.

**Fig. 3.**
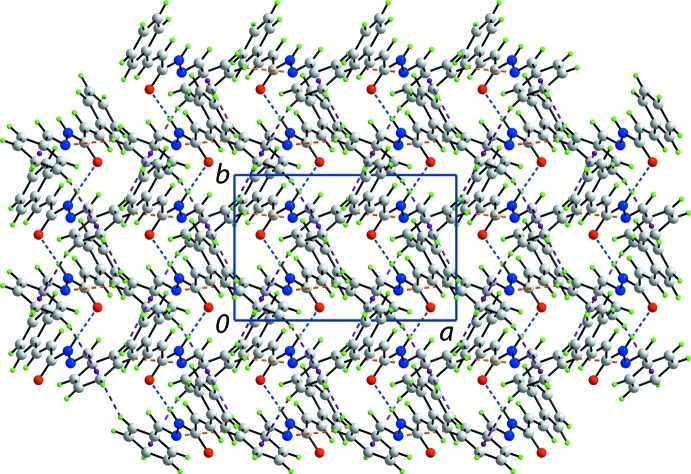
A view of the supramolecular layer in the ab plane in (I) sustained by N—H···O, C—H···N and C—H···π interactions, shown as blue, orange and purple dashed lines, respectively.

**Fig. 4.**
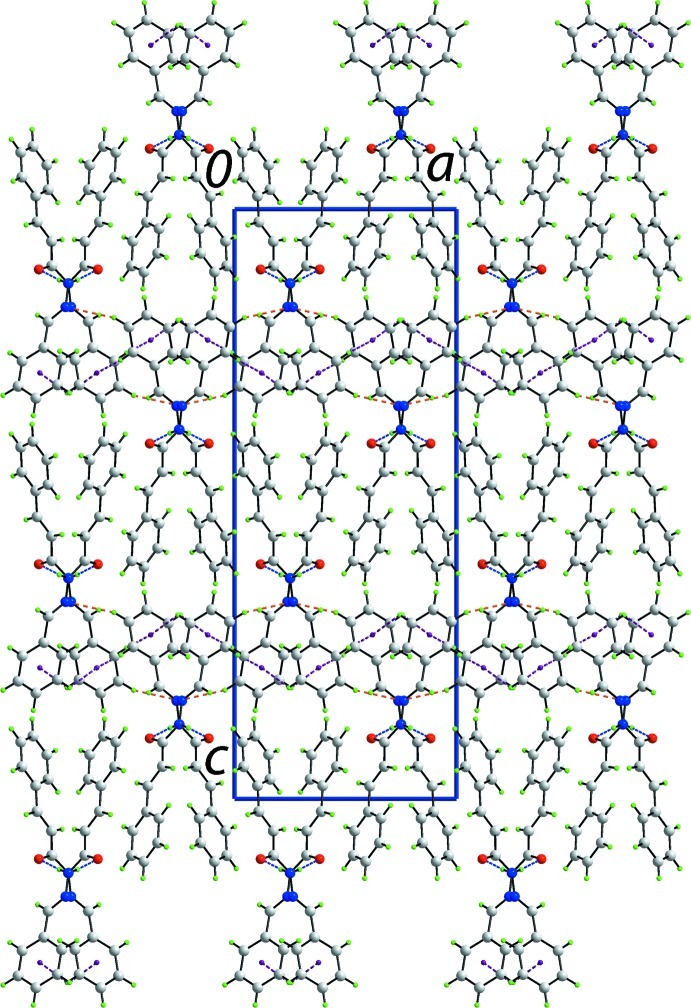
A view in projection down the b axis of the unit-cell contents for (I) showing the inter-digitation of layers. The N—H···O, C—H···N and C—H···π interactions, shown as blue, orange and purple dashed lines, respectively.

## supplementary crystallographic information

### Comment

(*E*)-Cinnamoylhydrazone derivatives have recently been shown to be agents against Chagas' disease (CD) (Carvalho *et al.*, 2012*b*), caused by the parasite *Trypanosoma cruzi*. CD is the major cause of infectious cardiopathy and represents an important public health problem. It affects approximately eight million people in Latin America (Rassi *et al.*, 2010). Neither the two established drugs, Nifurtimox and Benznidazole, is ideal because they present variable results depending on the phase of the disease, the dose and duration of the treatment, the patient's age and endemic region, as well as showing undesirable secondary side-effects (Soeiro & de Castro, 2011). The ArCH═ CHCONHN═CHAr' compounds used in the trypanocidal study (Carvalho *et al.*, 2012*b*) indicated considerable biological potential. Following on from our structural studies on (*E*)-PhCH═CH-CONHNHPh (Carvalho *et al.*, 2009), (*E*)-4-O_2_NC_6_H_4_CH═CH-CONHNHCOPh (Carvalho *et al.*, 2010*a*) and (*E*)-PhCH═CH-CONHN═ CHC_6_H_4_Cl-4.monohydrate (Carvalho *et al.*, 2010*b*), we now wish to report the crystal structure of one of the compounds from the trypanocidal study, namely the title compound, (I).

In (I), Fig. 1, there is a twist in the molecule as seen in the dihedral angle between the phenyl rings of 25.48 (12)°. The greatest deviation from a planar torsion angle is found for C2—C1—C7—N1 of 14.0 (3)°. The conformation about each of the imine [N1═C7 = 1.269 (3) Å] and ethylene [C9═C10 = 1.313 (3) Å] bonds is *E*. In the structure of the 4-chlorobenzylidene derivative (Carvalho *et al.*, 2010*b*), a decidedly more planar arrangement was noted (r.m.s. deviation of the 20 non-H atoms = 0.172 Å). However, a more twisted arrangement was found in the 2-hydroxyl derivative (Carvalho *et al.*, 2012*a*) where the dihedral angle between the benzene rings is 16.67 (8)°.

In the crystal of (I), the molecules are linked by N—H···O hydrogen bonds (Table 1), resulting in zigzag chains along the *b* axis. The chains are linked into a supramolecular layer in the *ab* plane by C—H···N and C—H···π interactions, Fig. 3 and Table 1; the layers inter-digitate along the *c* axis, Fig. 4.

### Experimental

The title compound was prepared as reported (Carvalho *et al.*, 2012*b*). The sample used in the crystallographic study was grown from its EtOH solution in the form of small colourless needles.

### Refinement

The C-bound H atoms were geometrically placed (C—H = 0.95 Å) and refined as riding with *U*_iso_(H) = 1.2*U*_eq_(C). The O– and N-bound H atoms were located from a difference map and refined with the distance restraints O—H = 0.84±0.01 and N—H = 0.88±0.01 Å, and with *U*_iso_(H) = *zU*_eq_(carrier atom); *z* = 1.5 for O and *z* = 1.2 for N.

### Crystal data

**Table:**

C_16_H_14_N_2_O	*F*(000) = 1056
*M**_r_* = 250.30	*D*_x_ = 1.266 Mg m^−^^3^
Orthorhombic, *P**b**c**a*	Synchrotron radiation, λ = 0.6943 Å
Hall symbol: -P 2ybc	Cell parameters from 996 reflections
*a* = 11.473 (19) Å	θ = 3.2–25.1°
*b* = 7.507 (13) Å	µ = 0.04 mm^−^^1^
*c* = 30.50 (5) Å	*T* = 120 K
*V* = 2627 (8) Å^3^	Needle, colourless
*Z* = 8	0.12 × 0.03 × 0.02 mm

### Data collection

**Table:**

Bruker SMART APEXII CCD diffractometer	1442 reflections with *I* > 2σ(*I*)
Radiation source: Daresbury SRS station 9.8	*R*_int_ = 0.084
Silicon 111 monochromator	θ_max_ = 22.7°, θ_min_ = 2.6°
fine–slice ω scans	*h* = −12→12
14542 measured reflections	*k* = −8→8
1876 independent reflections	*l* = −33→33

### Refinement

**Table:**

Refinement on *F*^2^	Primary atom site location: structure-invariant direct methods
Least-squares matrix: full	Secondary atom site location: difference Fourier map
*R*[*F*^2^ > 2σ(*F*^2^)] = 0.049	Hydrogen site location: inferred from neighbouring sites
*wR*(*F*^2^) = 0.140	H atoms treated by a mixture of independent and constrained refinement
*S* = 1.05	*w* = 1/[σ^2^(*F*_o_^2^) + (0.0776*P*)^2^ + 0.9415*P*] where *P* = (*F*_o_^2^ + 2*F*_c_^2^)/3
1876 reflections	(Δ/σ)_max_ < 0.001
175 parameters	Δρ_max_ = 0.18 e Å^−^^3^
1 restraint	Δρ_min_ = −0.20 e Å^−^^3^

### Special details

**Table:**

Geometry. All s.u.'s (except the s.u. in the dihedral angle between two l.s. planes) are estimated using the full covariance matrix. The cell s.u.'s are taken into account individually in the estimation of s.u.'s in distances, angles and torsion angles; correlations between s.u.'s in cell parameters are only used when they are defined by crystal symmetry. An approximate (isotropic) treatment of cell s.u.'s is used for estimating s.u.'s involving l.s. planes.
Refinement. Refinement of *F*^2^ against ALL reflections. The weighted *R*-factor *wR* and goodness of fit *S* are based on *F*^2^, conventional *R*-factors *R* are based on *F*, with *F* set to zero for negative *F*^2^. The threshold expression of *F*^2^ > 2σ(*F*^2^) is used only for calculating *R*-factors(gt) *etc*. and is not relevant to the choice of reflections for refinement. *R*-factors based on *F*^2^ are statistically about twice as large as those based on *F*, and *R*- factors based on ALL data will be even larger.

### Fractional atomic coordinates and isotropic or equivalent isotropic displacement parameters (Å^2^)

**Table:**

	*x*	*y*	*z*	*U*_iso_*/*U*_eq_	
O1	0.38302 (13)	0.0885 (2)	0.60236 (5)	0.0381 (5)	
N1	0.23795 (16)	0.2057 (3)	0.66603 (6)	0.0349 (5)	
N2	0.25029 (17)	0.2886 (3)	0.62606 (6)	0.0371 (5)	
H2N	0.209 (2)	0.385 (2)	0.6201 (8)	0.045*	
C1	0.14001 (19)	0.2034 (3)	0.73493 (7)	0.0338 (6)	
C2	0.21991 (19)	0.0947 (3)	0.75586 (7)	0.0373 (6)	
H2	0.2886	0.0588	0.7409	0.045*	
C3	0.2010 (2)	0.0384 (3)	0.79800 (7)	0.0403 (6)	
H3	0.2554	−0.0392	0.8117	0.048*	
C4	0.1040 (2)	0.0931 (3)	0.82057 (8)	0.0413 (7)	
H4	0.0924	0.0559	0.8500	0.050*	
C5	0.0236 (2)	0.2021 (4)	0.80048 (8)	0.0412 (7)	
H5	−0.0442	0.2389	0.8159	0.049*	
C6	0.0418 (2)	0.2580 (3)	0.75771 (8)	0.0386 (6)	
H6	−0.0134	0.3341	0.7439	0.046*	
C7	0.15938 (19)	0.2706 (3)	0.69056 (7)	0.0353 (6)	
H7	0.1121	0.3649	0.6799	0.042*	
C8	0.3245 (2)	0.2246 (3)	0.59613 (7)	0.0357 (6)	
C9	0.32566 (19)	0.3248 (3)	0.55473 (7)	0.0344 (6)	
H9	0.2785	0.4282	0.5518	0.041*	
C10	0.3909 (2)	0.2735 (3)	0.52168 (7)	0.0374 (6)	
H10	0.4399	0.1736	0.5268	0.045*	
C11	0.39690 (19)	0.3527 (3)	0.47781 (7)	0.0366 (6)	
C12	0.3407 (2)	0.5093 (4)	0.46697 (8)	0.0447 (7)	
H12	0.2972	0.5710	0.4887	0.054*	
C13	0.3468 (2)	0.5770 (4)	0.42517 (9)	0.0501 (7)	
H13	0.3079	0.6851	0.4181	0.060*	
C14	0.4090 (2)	0.4885 (4)	0.39369 (8)	0.0505 (7)	
H14	0.4134	0.5354	0.3648	0.061*	
C15	0.4645 (2)	0.3332 (4)	0.40364 (8)	0.0498 (7)	
H15	0.5069	0.2716	0.3816	0.060*	
C16	0.4593 (2)	0.2651 (4)	0.44565 (8)	0.0437 (7)	
H16	0.4988	0.1574	0.4525	0.052*	

### Atomic displacement parameters (Å^2^)

**Table:**

	*U*^11^	*U*^22^	*U*^33^	*U*^12^	*U*^13^	*U*^23^
O1	0.0345 (9)	0.0435 (10)	0.0362 (9)	0.0027 (8)	0.0002 (7)	0.0019 (8)
N1	0.0314 (10)	0.0474 (13)	0.0258 (10)	−0.0017 (9)	−0.0002 (8)	0.0017 (9)
N2	0.0373 (11)	0.0464 (14)	0.0276 (10)	0.0004 (9)	0.0001 (9)	0.0051 (9)
C1	0.0332 (12)	0.0437 (14)	0.0246 (12)	−0.0079 (11)	−0.0008 (10)	−0.0013 (10)
C2	0.0323 (13)	0.0443 (14)	0.0353 (13)	0.0027 (11)	0.0020 (10)	−0.0037 (11)
C3	0.0409 (14)	0.0453 (16)	0.0345 (13)	0.0002 (12)	−0.0052 (11)	0.0027 (11)
C4	0.0493 (16)	0.0497 (16)	0.0249 (12)	−0.0115 (13)	−0.0027 (11)	0.0007 (11)
C5	0.0339 (13)	0.0536 (17)	0.0362 (14)	−0.0068 (12)	0.0071 (11)	−0.0074 (12)
C6	0.0351 (13)	0.0428 (14)	0.0379 (14)	−0.0004 (11)	−0.0053 (11)	−0.0002 (11)
C7	0.0324 (13)	0.0423 (15)	0.0311 (13)	−0.0003 (11)	−0.0039 (10)	−0.0005 (10)
C8	0.0314 (12)	0.0457 (16)	0.0301 (13)	−0.0062 (12)	−0.0013 (10)	−0.0023 (11)
C9	0.0322 (12)	0.0411 (14)	0.0297 (12)	0.0003 (10)	−0.0021 (10)	0.0020 (10)
C10	0.0353 (13)	0.0439 (15)	0.0330 (13)	−0.0003 (11)	−0.0017 (11)	0.0015 (11)
C11	0.0315 (12)	0.0504 (16)	0.0279 (12)	−0.0045 (12)	−0.0002 (10)	0.0028 (11)
C12	0.0419 (14)	0.0579 (17)	0.0344 (14)	0.0029 (13)	0.0071 (11)	0.0013 (13)
C13	0.0423 (15)	0.0629 (19)	0.0449 (16)	0.0034 (13)	−0.0023 (12)	0.0127 (14)
C14	0.0398 (14)	0.081 (2)	0.0312 (14)	−0.0062 (14)	−0.0015 (11)	0.0134 (14)
C15	0.0394 (14)	0.077 (2)	0.0332 (14)	0.0000 (14)	0.0081 (11)	−0.0064 (14)
C16	0.0389 (14)	0.0530 (16)	0.0391 (15)	0.0035 (12)	0.0008 (11)	0.0019 (12)

### Geometric parameters (Å, º)

**Table:**

O1—C8	1.238 (3)	C7—H7	0.9500
N1—C7	1.269 (3)	C8—C9	1.470 (4)
N1—N2	1.376 (3)	C9—C10	1.313 (3)
N2—C8	1.338 (3)	C9—H9	0.9500
N2—H2N	0.886 (10)	C10—C11	1.466 (4)
C1—C2	1.383 (3)	C10—H10	0.9500
C1—C6	1.386 (4)	C11—C12	1.381 (4)
C1—C7	1.461 (4)	C11—C16	1.381 (4)
C2—C3	1.370 (4)	C12—C13	1.374 (4)
C2—H2	0.9500	C12—H12	0.9500
C3—C4	1.371 (4)	C13—C14	1.368 (4)
C3—H3	0.9500	C13—H13	0.9500
C4—C5	1.377 (4)	C14—C15	1.363 (4)
C4—H4	0.9500	C14—H14	0.9500
C5—C6	1.386 (4)	C15—C16	1.381 (4)
C5—H5	0.9500	C15—H15	0.9500
C6—H6	0.9500	C16—H16	0.9500
			
C7—N1—N2	115.0 (2)	O1—C8—C9	123.4 (2)
C8—N2—N1	120.5 (2)	N2—C8—C9	114.1 (2)
C8—N2—H2N	119.7 (17)	C10—C9—C8	120.9 (2)
N1—N2—H2N	119.8 (17)	C10—C9—H9	119.5
C2—C1—C6	118.8 (2)	C8—C9—H9	119.5
C2—C1—C7	122.0 (2)	C9—C10—C11	127.5 (3)
C6—C1—C7	119.1 (2)	C9—C10—H10	116.3
C3—C2—C1	120.6 (2)	C11—C10—H10	116.3
C3—C2—H2	119.7	C12—C11—C16	118.5 (2)
C1—C2—H2	119.7	C12—C11—C10	122.8 (2)
C2—C3—C4	120.5 (2)	C16—C11—C10	118.7 (3)
C2—C3—H3	119.8	C13—C12—C11	120.9 (2)
C4—C3—H3	119.8	C13—C12—H12	119.6
C3—C4—C5	119.9 (2)	C11—C12—H12	119.6
C3—C4—H4	120.1	C14—C13—C12	119.8 (3)
C5—C4—H4	120.1	C14—C13—H13	120.1
C4—C5—C6	119.8 (2)	C12—C13—H13	120.1
C4—C5—H5	120.1	C15—C14—C13	120.2 (3)
C6—C5—H5	120.1	C15—C14—H14	119.9
C1—C6—C5	120.3 (2)	C13—C14—H14	119.9
C1—C6—H6	119.8	C14—C15—C16	120.2 (3)
C5—C6—H6	119.8	C14—C15—H15	119.9
N1—C7—C1	121.5 (2)	C16—C15—H15	119.9
N1—C7—H7	119.3	C15—C16—C11	120.3 (3)
C1—C7—H7	119.3	C15—C16—H16	119.8
O1—C8—N2	122.5 (2)	C11—C16—H16	119.8
			
C7—N1—N2—C8	−175.3 (2)	O1—C8—C9—C10	−0.3 (4)
C6—C1—C2—C3	1.6 (4)	N2—C8—C9—C10	−177.4 (2)
C7—C1—C2—C3	178.0 (2)	C8—C9—C10—C11	176.4 (2)
C1—C2—C3—C4	−1.9 (4)	C9—C10—C11—C12	7.2 (4)
C2—C3—C4—C5	1.6 (4)	C9—C10—C11—C16	−171.3 (2)
C3—C4—C5—C6	−0.9 (4)	C16—C11—C12—C13	−0.2 (4)
C2—C1—C6—C5	−0.9 (3)	C10—C11—C12—C13	−178.7 (2)
C7—C1—C6—C5	−177.4 (2)	C11—C12—C13—C14	0.2 (4)
C4—C5—C6—C1	0.6 (4)	C12—C13—C14—C15	0.2 (4)
N2—N1—C7—C1	−176.63 (19)	C13—C14—C15—C16	−0.6 (4)
C2—C1—C7—N1	14.0 (3)	C14—C15—C16—C11	0.6 (4)
C6—C1—C7—N1	−169.6 (2)	C12—C11—C16—C15	−0.2 (4)
N1—N2—C8—O1	1.4 (3)	C10—C11—C16—C15	178.4 (2)
N1—N2—C8—C9	178.50 (19)		

### Hydrogen-bond geometry (Å, º)

Cg1 is the centroid of the C1–C6 benzene ring.

**Table:**

*D*—H···*A*	*D*—H	H···*A*	*D*···*A*	*D*—H···*A*
N2—H2n···O1^i^	0.88 (2)	1.93 (2)	2.816 (6)	175 (2)
C5—H5···N1^ii^	0.95	2.57	3.433 (7)	151
C3—H3···*Cg*1^iii^	0.95	2.92	3.618 (7)	131
C6—H6···*Cg*1^iv^	0.95	2.75	3.645 (7)	158

Symmetry codes: (i) −*x*+1/2, *y*+1/2, *z*; (ii) *x*−1/2, *y*, −*z*+3/2; (iii) *x*, −*y*−3/2, *z*−1/2; (iv) *x*+1/2, −*y*+1/2, −*z*+1.
